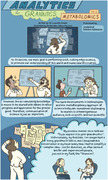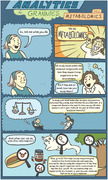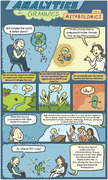# Analytics for Grannies 001: Metabolomics

**DOI:** 10.1002/ansa.202200901

**Published:** 2022-11-08

**Authors:** Tal Luzzatto Knaan

**Affiliations:** ^1^ Department of Marine Biology, The Leon H. Charney School of Marine Sciences University of Haifa 199 Aba Koushy Ave., Mount Carmel Haifa 3498838 Israel